# Enhancing communication, informed consent and recruitment in a paediatric urgent care surgical trial: a qualitative study

**DOI:** 10.1186/s12887-020-02040-w

**Published:** 2020-03-30

**Authors:** Frances C. Sherratt, Lucy Beasant, Esther M. Crawley, Nigel J. Hall, Bridget Young

**Affiliations:** 1 Institute of Population Health Sciences, University of Liverpool, Room 223, Second Floor, Block B, Waterhouse Building, 1-5 Dover Street, Liverpool, L3 5DA UK; 2 Bristol Medical School, University of Bristol, Bristol, UK; 3 University Surgery Unit, Faculty of Medicine, University of Southampton, Southampton, UK

**Keywords:** (3–10) qualitative, Randomised controlled trials, Communication, Appendicitis, Appendicectomy, Pediatric, Surgery, Urgent care, Emergency, Interviews

## Abstract

**Background:**

Recruiting patients to paediatric trials can be challenging, especially in trials that compare markedly different management pathways and are conducted in acute settings. We aimed to enhance informed consent and recruitment in the CONTRACT trial (CONservative TReatment of Appendicitis in Children a randomised controlled Trial; ISRCTN15830435) – a feasibility trial that compared non-operative treatment (antibiotics) versus appendicectomy for uncomplicated acute appendicitis.

**Methods:**

Qualitative study embedded within CONTRACT and conducted across three UK children’s hospitals. Data were transcribed audio-recordings of 85 CONTRACT recruitment consultations with 58 families; and semi-structured interviews with 35 health professionals and 28 families (34 parents, 14 children) invited to participate in CONTRACT. Data analysis drew on thematic approaches. Throughout CONTRACT, we used findings from the ongoing qualitative analysis to inform bespoke communication training for health professionals recruiting to CONTRACT. Before and after training we also examined qualitative changes in communication during consultations and quantitative changes in recruitment rates.

**Results:**

Bespoke communication training focussed on presenting the trial arms in a balanced way, emphasising clinical equipoise, exploring family treatment preferences and managing families’ expectations about the trial’s treatment pathways. Analysis of recruitment consultations indicated that health professionals’ presentation of treatment arms became increasingly balanced following training, (e.g. avoiding imbalanced terminology) and recruitment rose from 38 to 62%. However, they remained reluctant to explore families’ treatment preferences and respond with further information to balance these preferences. Analyses of interviews identified the time constraints of the urgent care setting, concerns about coercion, and reservations about exposing children to conversations about treatment risks as reasons for this reluctance. Interviews with families indicated the importance of clear explanations of trial treatment timings and sensitive communication of treatment allocation for both recruitment and retention.

**Conclusions:**

Following bespoke training based on the qualitative analyses, health professionals presented CONTRACT to families in clearer and more balanced ways and this was associated with an increase in the recruitment rate. Despite training, health professionals remained reluctant to explore families’ treatment preferences. We provide several recommendations to enhance communication, informed consent, recruitment and retention in future trials in urgent care settings.

## Background

Recruitment of patients to clinical trials is often sub-optimal [1], resulting in underpowered trials and to promising interventions being abandoned or delayed [2]. Recruiting children and young people to trials can be especially challenging [3], with the need to consider the perspectives of both child and parent [4] and that a child’s capacity varies substantially according to age and maturity [5]. Recruiting to trials that compare markedly different treatment arms, such as surgical and non-surgical treatments, is also known to be difficult as patients and health professionals often have strong preferences for a particular treatment [6, 7]. Recruiting to trials during an unscheduled hospital admission, and in settings where the investigational treatments need to be delivered urgently, presents further complexities given uncertainties regarding the patient’s clinical condition, coupled with limited time to recruit patients [8].

All these recruitment challenges were pertinent to the CONTRACT trial (CONservative TReatment of Acute Appendicitis in Children: a randomised controlled Trial). This was a feasibility randomised controlled trial comparing non-operative treatment (involving antibiotic treatment but no operation) with appendicectomy in children and young people with uncomplicated acute appendicitis [9]. The surgical treatment arm in CONTRACT has been a mainstay of treatment for acute appendicitis for over 100 years [10], so we anticipated that health professionals and families would have strong preferences for a surgical intervention. Additionally, patients eligible for CONTRACT have an acute illness and often present outside of normal working hours when recruiting staff availability is limited. Due to these concerns and limited UK data on the clinical effectiveness of non-operative treatment arms, we first designed and conducted the CONTRACT feasibility trial ahead of a planned full efficacy trial.

Increasingly, researchers are embedding qualitative studies in trials to identify barriers to recruitment and retention, and implement strategies to overcome these [11, 12]. Such qualitative studies can be especially valuable when embedded in feasibility trials to optimise design and conduct prior to a future definitive trial [13]. Qualitative research has identified several strategies to optimise recruitment by enhancing communication about trials. These include avoiding misinterpreted terms, eliciting, exploring and balancing patient treatment preferences [14–17], and identifying and addressing a lack of clinical equipoise among health professionals [18]. Such strategies help to avoid patients’ decisions about participation in trials being founded on misconceptions about treatment arms, therefore enhancing informed consent and recruitment [15, 16].

Most qualitative studies embedded in trials have focused on optimising trials involving adult patients. We embedded a qualitative study (the Communication Study) within CONTRACT, a children’s trial. Drawing on this embedded study’s findings regarding barriers to recruitment in CONTRACT, we then developed and delivered bespoke training for recruiters to enhance informed consent and recruitment as CONTRACT was ongoing. We examined qualitative changes in health professionals’ communication before and after the bespoke training, and changes in the rates of recruitment to CONTRACT. In this paper, we report on the broad lessons from the Communication Study to help trialists enhance informed consent and recruitment in future paediatric surgical trials in urgent care settings.

## Methods

### Overview

**Fig. 1:**
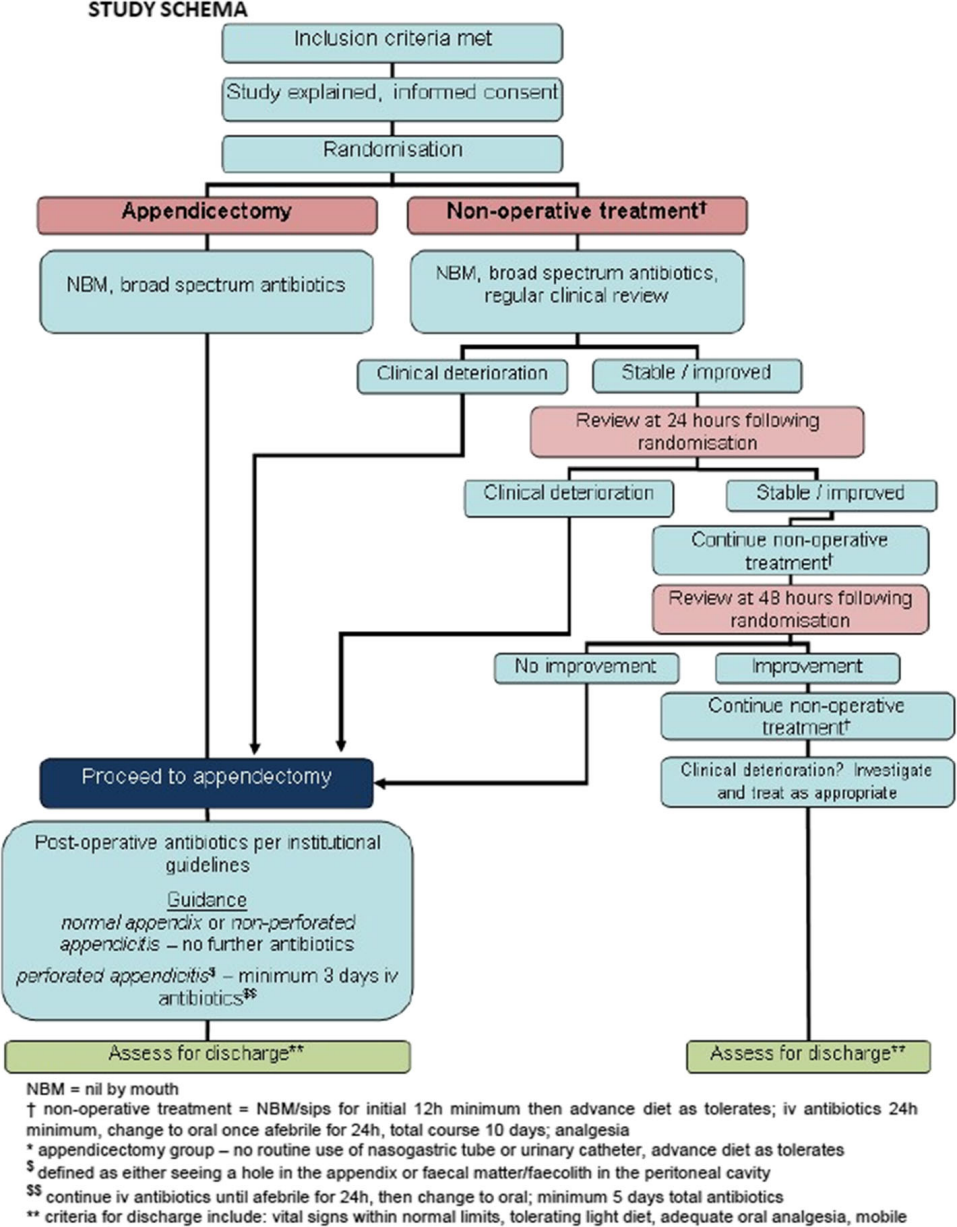
Summary of the patient pathway in the CONTRACT feasibility trial

This qualitative study, known as the Communication Study, was embedded in CONTRACT, a randomised feasibility trial to inform a future definitive trial comparing appendicectomy versus non-operative in children and young people with uncomplicated acute appendicitis [9]. Figure 1 provides an overview of the patient pathway in CONTRACT.

Drawing on previously reported methods [19], we collected and qualitatively analysed audio-recordings of CONTRACT consultations and semi-structured interviews with patients, parents of patients and health professionals. Consultation recordings allowed us to explore how health professionals communicated about CONTRACT with families during recruitment consultations, whilst interviews allowed us to explore the perspectives of children, parents and health professionals on communication during recruitment. The Communication Study was included in CONTRACT’s ethical approval (South Central Hampshire A, National Health Service Research Ethics Committee, ref.: 16/SC/0596).

### Participants

Between March 2017 and February 2018, within all three CONTRACT sites (which were UK hospital emergency departments and acute admission wards), health professionals approached families of eligible children, inviting them to take part in CONTRACT and the Communication Study. Families could participate in CONTRACT, the Communication Study (CONTRACT consultation recording and/or interview), both or neither. Parents were invited for interview if they had been approached about CONTRACT; children aged 7–15 years who had been approached about CONTRACT were also invited for interview. Health professionals were invited for interview if they had either approached families about CONTRACT or were involved in recruitment or patient care. We monitored sampling to ensure we included families who declined CONTRACT as well as those who consented, and to encompass variability in child age, family socio-economic status and hospital sites. We also monitored sampling for data saturation, the point at which new themes ceased being identified [20], although we continued sampling until close to the end of CONTRACT in order to examine any post-training changes in communication.

### Procedure

#### Consultations

Health professionals requested verbal permission to audio-record CONTRACT consultations immediately before the consultation, then sought written consent/assent from parents and children at the end of the consultation. Health professionals uploaded audio-recorded consultations and Communication Study consent/assent forms directly to the Communication Study team.

#### Semi-structured interviews

Families who provided written consent/assent for contact from the Communication Study team were telephoned by a team member who explained the study, forwarded the interview information sheet and provisionally scheduled an interview with willing families. Informed consent was obtained prior to interview. Interviews were typically 1–4 weeks following discharge from hospital.

The Communication Study team typically contacted health professionals via the local principal investigator to invite them to be interviewed. Informed consent was obtained before health professionals were interviewed.

Two experienced female qualitative researchers (LB and FS) with health research backgrounds, conducted all interviews either face-to-face or by telephone. Interviews were topic-guided to ensure exploration of key topics (see Table 1), yet conversational to allow participants to raise issues of importance to them. Separate topic guides were devised for parents, health professionals and children and young people; FS and LB used art pads, colouring pens and stickers to facilitate the children’s interviews. A study advisory group, comprising children and young people with experience of appendicitis or with an interest in research, and their parents, informed the development of the topic guides and these were adapted throughout the study.

**Table 1:** Key topics explored in the child, parent and health professional interviews

**Children and parent interviews**	
• Experience of illness	
• Initial thoughts about CONTRACT	
• Experience of being approached about CONTRACT	
- Thoughts on how CONTRACT was explained	
- How the health professional explained the treatment options	
- Family preferences	
- Recollection of key aspects of CONTRACT	
• Decision-making about CONTRACT participation/non-participation	
• Views and understanding of randomisation	
• Experience of treatment	
• Experience of recovery	
• Reflections on CONTRACT since being approached	
**Health professional interviews**	
• Initial thoughts about CONTRACT	
• Knowledge of CONTRACT and views on its aims	
• Recruitment pathways	
• Experiences of approaching families	
• Health professional treatment preferences	
• Experience of delivering the treatments	
• Anticipated CONTRACT results	

### Analysis

Analysis of pseudo-anonymised audio-recorded consultations and interviews drew on thematic analysis [21] and several other methodological traditions, comparing both across data types (i.e. family member interviews, health professional interviews or consultations) and within cases (i.e. matched family member and health professional interviews, and consultation[s]).

LB and FS initially read transcripts of consultations and interviews, ‘cycling’ between the developing analysis and new data. LB and FS developed open codes, which they organised into frameworks to code and index the transcripts using QSR NVivo 11 [22]. They double-coded approximately 10% of transcripts, reviewing this to ensure consistency. BY also read a selection of transcripts, while several members of the wider team (LB, FS, EC, NH and BY) met periodically to discuss and ‘test’ the developing analysis. If analyses identified communication during consultations that was unclear or likely to deter informed consent or recruitment, the Communication Study team integrated it into the health professional training sessions (see further details of training below).

We provide illustrative quotes in the results section below labelled by: data type (Cons = Consultation, Int = Interview; participant roles/relationships (Surgeon, Nurse, Mother, Father, Child); family code number and CONTRACT treatment allocation and/or participation status (NOT = Non-operative treatment, App = Appendicectomy, Declined = Declined, Withdrew = Withdrew). We also indicate each health professional with a number to aid the reader in linking their consultations with interviews. Children’s ages are shown with their quotes. Of note, in the quotations below, participants frequently refer to the non-operative treatment arm as the ‘antibiotic’ arm.

### CONTRACT communication training

In December 2016 (pre-CONTRACT), informed by the previous literature [12, 14–16] we delivered generic communication training to health professionals who would likely be approaching families about CONTRACT at each site. The subsequent bespoke training was additionally informed by the ongoing qualitative analysis as outlined above. We structured the analysis and the delivery of the bespoke training by dividing the CONTRACT recruitment period into three phases - phase one (months 1–4), phase two (months 5–8), and phase three (months 9–12). At each CONTRACT site, we delivered the bespoke training sessions at the start of phase two (July 2017) and phase three (November 2017). These training sessions were discursive and informal with the Communication Study team presenting the recruitment data, anonymised excerpts from the consultation and interview data, whilst health professionals reflected on their approach to communication. We also provided health professionals with ‘hints and tips’ sheets on optimising communication about CONTRACT, and we periodically updated these in response to progress with CONTRACT and ongoing analysis of the qualitative data.

## Results

### Communication study dataset characteristics

**Fig. 2:**
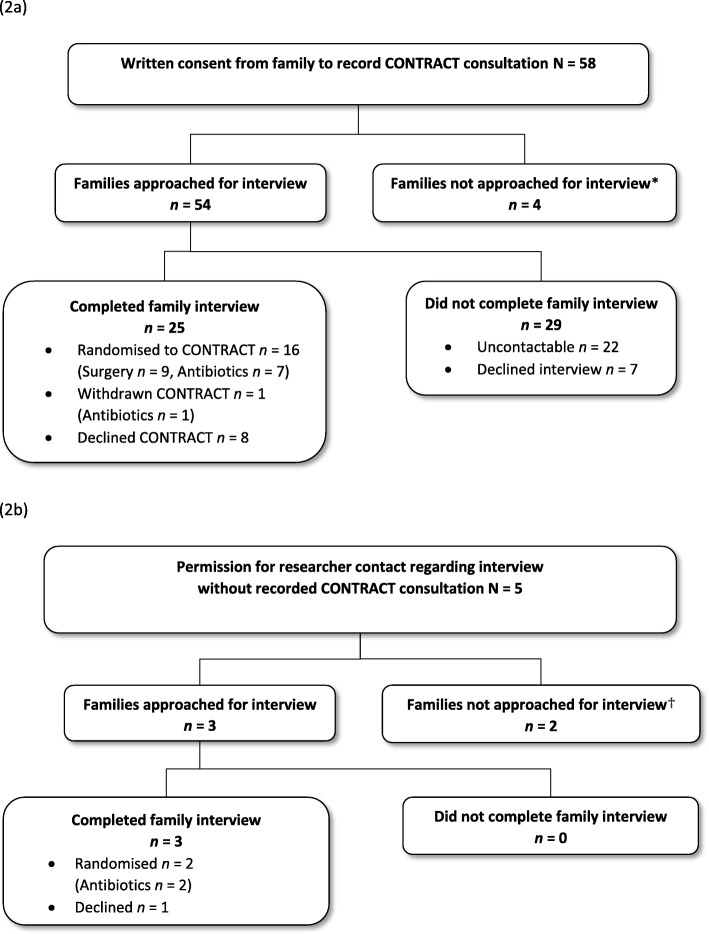
Participation in the Communication Study

Figure 2a and b provide an overview of recruitment of families (both those with recorded CONTRACT consultations and those without recorded CONTRACT consultations), showing families’ trajectories through CONTRACT and the Communication Study. Of the 115 families who were approached about CONTRACT across three sites, health professionals obtained informed consent from 58 (50%) families to audio-record recruitment consultations and from 62 (54%) families to be contacted regarding a qualitative interview. In total, we had 85 audio-recorded CONTRACT consultations from 58 families, and completed 28 family interviews, and 40 interviews with 35 health professionals. Families were spread relatively evenly across the sites and from diverse socio-economic backgrounds. Table 2 provides further details of participant and Communication Study data characteristics.

**Table 2:** Participant and Communication Study data characteristics

Families who provided a consultation recording	*N* = 58
Total consultations recorded	85
Initial (median duration in minutes, range)	58 (10, 1–24)
Subsequent including second, third and/or fourth	27
CONTRACT participation status	
Consent (v decline)	38 (v 20)
Treatment allocation	
Non-operative (v appendicectomy)	19 (v 19)
Patient characteristics	
Median age (range)	10 (4–15)
Males (v females)	39 (v 19)
Families interviewed	*N* = 28
Interview median duration in minutes (range)	59 (22–89)
Format of interview	
Face-to-face (v telephone)	12 (v 16)
Patient characteristics	
Median age (range)	11 (5–15)
Males (v females)	21 (v 7)
Health professionals interviewed	*N* = 35
Total interviews recorded	40
Initial (median duration in minutes, range)	35 (48, 20–79)
Subsequent (median duration in minutes, range)	5 (51, 39–69)
Health professional’s role	
Surgeon	25
Research nurse	7
Ward nurses	3
Format of interview	
Face-to-face (v telephone)	23 (v 17)

Most parents (*n* = 19/28, 68%) completed an interview without their child being present. Fifteen interviews were completed with mothers only, seven with fathers only, and six with both parents present. We interviewed 14 children.

### Identifying opportunities to enhance informed consent and recruitment

Consultations typically entailed health professionals describing elements of CONTRACT and the Communication Study, providing relevant information sheet(s) and showing a video about CONTRACT. Across the three recruitment phases, CONTRACT recruitment rates rose from 38% in phase one, to 50% in phase two and 62% in phase three. Parents tended to prefer surgery over non-operative treatment and those with such preferences were usually less willing to participate in CONTRACT. They often had previous experience of perforated or complicated appendicitis in themselves or a family member, and had concerns that non-operative treatment would not work or that the appendicitis might recur. In contrast, children tended to fear surgery and prefer non-operative treatment. In the following sections we describe how health professionals communicated about CONTRACT during consultations, and family and health professional experiences of communication and of CONTRACT more broadly. We also describe how the qualitative findings informed the bespoke communication training sessions that we delivered while CONTRACT was ongoing, and outline qualitative changes in patterns of health professionals’ communication across phases one, two and three.

#### Imbalanced content and presentation of trial arms

##### Imbalanced language

In their interviews, families generally described positive experiences of communication about CONTRACT. However, analysis of phase one consultations showed that health professionals often referred to treatment arms, particularly surgery, using terms that implied it was superior to non-operative treatment. For example, they referred to surgery as the “*gold standard*” or “*normal pathway*”, while referring to non-operative treatment as “*experimental*” or “*just antibiotics*”. In both bespoke training sessions we fed back these findings. We discussed the advantages of using neutral, non-evaluative terms for surgery, such as “*operation*” or “*surgery treatment*”, and similarly for non-operative treatment, we discussed simply referring to “*antibiotic treatment*” or “*medicine*”. Analysis of consultation data following the phase two and three bespoke training sessions indicated that health professionals became more balanced in the terms they used to describe treatment arms and used fewer imbalanced terms.

In phase one, some health professionals inadvertently suggested that CONTRACT participation could be burdensome for either the family or the clinical team: *“[If] you decide ‘oh no, I don’t want to have all of this done, I don’t want to go to all this trouble’… our standard way would be at the moment is to go for an operation*”. (Cons_Surgeon33_Family15_Declined). We fed this back to health professionals through the bespoke communication training. In phases two and three we found that health professionals mostly avoided statements that CONTRACT could be burdensome, and increasingly framed CONTRACT positively.

##### Exploring family treatment preferences and balancing trial arms

In phase one we found that health professionals rarely asked questions to elicit or explore family treatment preferences. Some families did spontaneously voice their preferences, but health professionals mostly took these at face value and did not explore further or attempt to balance families’ preferences:

*Surgeon 7: Do you want to know a bit more about it [CONTRACT]?*

*Mother 6: Um, I don’t think... no, I’d just rather get…*

*Surgeon 7: You’d just rather get on?*

*Mother 6: Yeah, the normal way.*

*Surgeon 7: Okay, that’s absolutely fine. Um, so in that case, what we’ll try to do is take his appendix out, okay.*

(Cons_Surgeon7_Family6_Declined)

While some health professionals did provide information to balance families’ views about treatments, they did not explore the underlying reasons for families’ treatment preferences. In the bespoke communication training sessions we described the steps involved in exploring families’ treatment preferences, including identifying preferences, exploring the reasons for preferences, and gently challenging and balancing families’ preferences. We presented excerpts from families whose preferences for surgery were based on their experiences of perforated or complicated appendicitis, rather than uncomplicated acute appendicitis. We encouraged health professionals to explore such preferences further, and where appropriate, explain the differences between perforated/complicated appendicitis and uncomplicated acute appendicitis, so highlighting treatment equipoise.

Following this training on preference exploration, we found some changes to consultations in phases two and three. For example, health professionals started to ask more specific questions to elicit treatment preferences: *“Is there anything you think about that is sort of, the idea of being involved in research, something that appeals to, that sort of worries you?”* (Cons_Surgeon29_Family17_App). We also found more examples of health professionals gently exploring preferences and providing balanced information about treatment arms, although these remained relatively infrequent throughout phases two and three. In interviews, while some health professionals described the benefits of exploring treatment preferences with families, others expressed concerns. These included that balancing family preferences for non-operative treatment (e.g. by detailing surgical risks) could unduly worry some families, and that exploring preferences could either be viewed by families as coercive or dissuade families from participating if they had a preference for non-operative treatment:

*It's difficult when you're just trying to get people into the study… if the situation arose again and there was some situation where they were… very pro … the non-operative arm, then that would have been an opportunity to, to go through that. But at that point, you know, it's a success, it's a tick in the success column, we just take it and run.* (Int_Surgeon57)

*What I didn’t want to do was to be the person who pushes it too much and they complain.* (Int_Surgeon18)

Some surgeons indicated that they provided a ‘distilled’ description of surgical risks, to avoid unduly worrying families: “*I don’t say it in such frank, scary terms but I say, you know, if you have an operation, you might come back at some point in the next year or two with a complication from the surgery.*” (Int_Surgeon10). Some also said that they would discuss surgical risks in detail only with parents who wanted to discuss them: “*In the parents who want to talk about it at length, which I’ve had a few of, then I would explain that to them*” (Int_Surgeon37).

##### Health professional clinical equipoise

Throughout all phases, health professionals typically provided families with a clear rationale for CONTRACT, explaining the uncertainty regarding treatment for children with uncomplicated acute appendicitis: **“***What we are doing is looking at whether treating appendicitis with, um, an operation, or if you can avoid an operation and treat it with just antibiotics*” (Cons_Surgeon29_Family25_Declined). In interviews, most health professionals commented that CONTRACT addressed an important research question: “*I felt that this is a really important thing to be doing, because it’s in everybody’s interests to know if … we can treat appendicitis with antibiotics in the future*” (Int_Surgeon40). Nevertheless, health professionals often made statements indicating their own treatment preferences and lack of equipoise in CONTRACT (see Table 3 for examples) noting that appendicectomy was the “*traditional*” treatment.

**Table 3:** Statements indicating health professionals’ lack of clinical equipoise in CONTRACT

Preference for appendicectomy	Preference for non-operative treatment
Surgery as standard care: *“I’ve been doing surgery now for 15 years, so appendicitis equals an operation and it’s quite difficult to change your mindset.”* (Int_Surgeon54)	Experience of antibiotics as effective: *“You watch some patients get better with antibiotics and it’s really, really tempting to just not sort of bother with the trial and just offer patients antibiotics occasionally, which I haven’t done. But, you know, it’s quite hard to sort of, you know, keep your own personal views under control as you see it unfold.”* (Int_Surgeon17)
Patient perceived as more poorly leading to doubts about eligibility: *“How they look and if they obviously look pretty sick, then I think you’ll be more reluctant to do something that doesn’t feel standard… He was definitely eligible, for sure. But… he looked like he had appendicitis which, which is not entirely well.”* (Int_Surgeon37)	Patient perceived as less poorly leading to doubts about eligibility: *“We do agree that for the selected group of patients [antibiotics] would work… The irony is that sometimes we have selected certain people that we think ‘oh, they definitely, it’s more the early appendicitis type and not the complicated appendicitis and would definitely do well’, but … sometimes you feel sad that someone that looked really well and would do really well with antibiotics alone, is then randomised to having an operation.”* (Int_Surgeon11)
Avoiding contributing towards antibiotic resistance: *“You could argue that more [families] than not will go towards the antibiotics rather than surgery. Unless of course you have more scare stories about how antibiotic resistance is coming in… that may well influence how people decide in the longer term.”* (Int_Surgeon12)	
Fewer surgical training opportunities: *“You take away these straightforward… training operations which can become useful … for people building basic skills... In the longer term you … have to become more inventive or find different ways … for people to gain their surgical experience and that could be a counter risk going forward.”* (Int_Surgeon12)	

Linked to this, health professionals often perceived some children to be particularly suitable for one treatment arm or the other. For example, children who were particularly poorly were perceived to be more suitable for surgery, whilst those who were relatively well were felt to be more suitable for non-operative treatment (see Table 2), despite both groups being eligible for CONTRACT according to the protocol. These concerns were usually borne out of surgeons’ worries about diagnosing children with uncomplicated acute appendicitis. A key inclusion criterion for CONTRACT was for children to have a clinical diagnosis (with or without radiological assessment) of acute appendicitis, which before CONTRACT commenced, would have been treated with appendicectomy. CONTRACT thus brought a new challenge for surgeons - distinguishing whether children had uncomplicated acute appendicitis or perforated appendicitis.

##### Describing randomisation

In phase one we found some issues with how health professionals communicated about randomisation in consultations with families. For example: “*we will actually go and put in a little bit of information about [child] into the computer and it will pick a treatment arm*” (Cons_Surgeon8_Family45_NOT). Interviews with families indicated that such explanations led them to think the computer selected the most appropriate treatment for their child: “*Once all the information had been gathered by the medics, it was being put into the computer… to see whether or not… he had to go down the, medical, the antibiotics route or the surgery route*” (Int_Mother48_NOT).

In the bespoke communication training sessions, we advised health professionals to avoid explanations that might imply that treatments in CONTRACT were allocated according to what might be suited to an individual child, and more generally, to be careful in referring to the use of computers in the randomisation process. In subsequent CONTRACT consultations, we found that some health professionals adjusted their explanations to avoid these problems: *“A computer is going to pick at random half the children to have an operation and half the children to have antibiotics, and it’s only by doing that that we can have two fairly distributed groups”* (Cons_Surgeon10_Family44_App).

#### Time pressures in urgent care trials

##### Managing families’ expectations about trial treatments

As noted previously, parents often expressed a preference for surgery over non-operative treatment and therefore declined CONTRACT. Typically, families preferred surgery because they believed it would avoid perforation and would give immediate pain relief. Given these preferences, in an initial effort to balance explanations, health professionals often made statements about non-operative treatment such as, “*if we’ve got any doubt that he needs an operation at any time, he can have an operation at any time*” (Cons_Surgeon10_Family47_NOT). However, health professionals rarely mentioned that it is not possible to guarantee timing of unscheduled surgery and that cases are prioritised based on clinical need. Interviews indicated that some families interpreted such comments to mean surgery would be undertaken immediately following an assessment showing that non-operative treatment had failed. In the bespoke communication training we encouraged health professionals to manage families’ expectations about the timing of surgery by clarifying how children were monitored and the timescale of surgery should non-operative treatment fail. In subsequent consultations we found that health professionals changed their communication in line with the training: *“We will monitor him, okay. And in the next 24 to 48 hours… If things do not get better, okay, or if he becomes worse… we will proceed with an operation … but it may take a few hours”* (Cons_Surgeon41_Family26_App).

##### Providing families with optimal time to decide

Families were often provided with several hours to deliberate about whether to participate in CONTRACT. This period of deliberation, while consistent with ethical guidance, meant families typically had a period of uncertainty regarding which treatment they were to be allocated to if they did wish to participate, or when treatment would commence if they did not wish to participate. Whilst, in interviews, most families suggested 1–2 h was a reasonable time frame to decide, some parents and children had “*decided straightaway”* (Int_Child57_Age12_NOT) and felt the time to decide was “too long” as they wanted to know which treatment they were going to receive*.*

Some families also reported that health professionals had delayed or withheld antibiotic treatment or pain relief until the family were able to voice their decision about CONTRACT participation. In these cases, families often questioned whether the study had adversely affected their child’s care: *“Did they delay the antibiotics… it seemed strange that the surgeon had told me earlier on in the day that they were gonna to start him on the IV antibiotics. But then he never started it until after we’d seen the, the lady surgeon from the research”* (Int_Father33_Declined).

##### Recruiting outside normal working hours

In interviews, several health professionals suggested that it was particularly challenging to approach and recruit families to CONTRACT at weekends, evenings and nights. This resulted in some eligible families not being approached about CONTRACT, although surgeons suggested this was rare. Surgeons explained that having research nurses available to support them, at least during normal working hours, was highly beneficial. Research nurses also explained that staff occasionally overlooked CONTRACT recruitment activities outside of normal hours: “*it has been missed giving them [families] the [CONTRACT] information sometimes*” (Int_Nurse2).

#### Challenges involving children and young people in decision-making

##### Children’s capacity to engage in research conversations

When interviewed several weeks after their treatment most children were able to recall that CONTRACT examined treatment of appendicitis with antibiotics. However, consultations and interviews indicated that children had often been in too much pain at the height of their illness to engage in the discussions and decision-making regarding CONTRACT:

*Surgeon 8: Did the video make any sense to you [child] or are you feeling a bit too sore?*

*Child 42: [Crying] … too sore.*

(Cons_Surgeon8_Child42_Age11_Declined)

*Child 33: It was hard for me to concentrate…*

*Mother 33: The lady was asking him questions, wasn’t she? And you were just going, ‘Oh I just want it, I just want to stop it’.*

(Int_Family33_Age12_Declined)

Therefore, with the exception of a few older patients, children tended to have little involvement in CONTRACT discussions. Enhancing children and young people’s involvement in decision-making in such settings will be challenging.

##### Discussing treatment risks with children

Some parents of younger children were concerned that discussing CONTRACT in front of children would or had made children more anxious. Parents were particularly concerned about their child hearing descriptions of the risks and benefits of CONTRACT treatments as a parent of a nine year old commented:

*When [the surgeon] went through all the complications… I even said to the doctor… “does he need to, does he really need to know this?” … when they’re in that much pain, and frightened anyway, I don’t think they need to know all of that… perhaps those conversations should be made outside the room, you know, away from the child.* (Int_Mother44_App)

##### Managing conflicting treatment preferences within families

In consultations and interviews, we often found that parents and children differed in their treatment preferences and in their willingness to participate in CONTRACT. Children tended to prefer non-operative treatment, whilst parents preferred surgery. Some families participated in CONTRACT despite such differences, with the preference of the child to participate usually taking precedence as one mother, who would have preferred for her child to have surgery rather than participate in CONTRACT commented to her child: “*I was respecting what you’d decided to do. You wanted to do the study”* (Int_Family57_Age12_NOT). In interviews, some surgeons spoke of randomisation within CONTRACT as offering a way of resolving the conflict within families:

*I use that [difference of opinion] as fuel to try and recruit them into the study… there's a disagreement here within the family, let's take it out of your hands as a family and, let the computer decide sort of thing.* (Int_Surgeon10)

#### Post-randomisation factors that may influence retention

##### Informing families of treatment allocation

In interviews, some families spoke of their disappointment on hearing that they had not been allocated to their favoured treatment. Some children even became upset: “*[Child] broke down [when he heard which treatment he was allocated to]… I think he was really gutted that it came up he needed surgery”* (Int_Mother36_App). One mother described being informed of the allocation to her non-preferred treatment preference in a brief and unfavourable manner:

*I was talking to a nurse… the consultant came round and said ‘no, sorry, she’s not got it’, I was like, ‘What? Not got what? What?’ So that was a bit of a blow. I think I’d rather have been told away from her [daughter]… that felt like it was thrown at me.* (Int_Mother32_App)

While most families continued in CONTRACT regardless of their treatment allocation, the one family we interviewed who withdrew from the trial did so because they had been randomised to their non-preferred treatment.

##### Post-surgical discussions

In interviews, several parents who had participated in CONTRACT commented that non-operative treatment would not have been effective in treating their child’s appendicitis. Their views seemed to be informed by post-operative discussions with surgeons. For parents of children who were randomised to non-operative treatment which subsequently failed, hearing details of the surgery post-operatively induced feelings of guilt:

*So she’d had all the delay with the drip, it didn’t work... I have felt a bit guilty that maybe if I’d have gone with my initial instinct, which was to just get the operation over and done with… that she might not have had it perforate.* (Int_Mother45_NOT)

Post-operative discussions also led some families to retrospectively question whether their child should have been eligible for CONTRACT. Such experiences may impede families’ trust in health professionals during trial follow-up and influence their compliance with trial follow-up activities.

## Discussion

This qualitative ‘Communication Study’ was embedded within the CONTRACT feasibility trial, with the aims of optimising CONTRACT communication and recruitment, as well as informing a future definitive trial. It is the first to report on analyses of trial consultations and interviews with health professionals, children and parents. By doing so, we were able to identify specific challenges to paediatric trials and propose strategies to optimise trial communication.

Informed by previous qualitative embedded studies, we identified key areas of non-optimal trial communication that can impede recruitment, such as the use of imbalanced terminology [14, 23] and a lack of treatment preference exploration [15, 16]. Following feedback in bespoke communication training sessions, health professionals reduced the use of imbalanced and confusing terminology. Recruitment rates also increased in the phases following the bespoke communication training. However, other aspects of health professionals’ communication, particularly preference exploration and balancing changed little despite training.

Treatment preference exploration has previously been found to optimise informed consent and recruitment [16, 18]. Although balancing treatment preferences is advocated in the literature, we identified distinctive complexities in doing so in a paediatric urgent care trial. Some health professionals remained particularly reluctant to explore families’ treatment preferences following training. They were concerned about unduly worrying families about treatment risks, believed that exploring treatment preferences was tantamount to coercing families to participate, or felt that exploring families’ preferences for non-operative treatment could dissuade them from participating in CONTRACT.

While most health professionals in interviews spoke about the value of the research question that CONTRACT aimed to address, similar to previous studies [18, 24], many had a strong preference for surgery. These biases were also apparent in the early phase recruitment consultations with families when health professionals used terms that were loaded in favour of one of the treatments, usually surgery. Health professionals’ lack of equipoise may also have added to their reluctance to explore treatment preferences and future research with families would help to establish how they experience treatment preference exploration and whether they also hold qualms about it.

Informed by the findings of the current study, we have developed recommendations to help enhance informed consent, recruitment and retention of families to future paediatric urgent care surgical trials (Table 4). The recommendations may be useful for paediatric trial recruitment more broadly.

**Table 4:** Recommendations to optimise informed consent and recruitment in paediatric urgent care surgical trials

(1) Present the trial arms in a balanced way in recruitment consultations, using neutral terminology and emphasise clinical equipoise. (2) Elicit and acknowledge family treatment preferences. Where possible, explore the reasons underlying these preferences and provide information to balance preferences and address any misconceptions. (3) Involve children and young people in research discussions and decision-making as consistent with guidance from the UK Nuffield Council on Bioethics [5]. This recommends that where possible, decisions about research should be shared decisions by children and their parents. It adds that “children should be as involved in decisions as they wish, and are able to be. Where children and young people have sufficient maturity and understanding, but are not yet treated legally as adults, professionals should seek consent both from children and from their parents.” (4) Some parents may be anxious about what their child hears about treatment procedures and risks. It is important to be sensitive to these anxieties when discussing a trial. (5) Provide families with advance information about how a child’s treatment will be managed pre-randomisation and in both treatment arms. Where relevant, this should include the timing of trial treatments and the timeframe in which families should expect to see an improvement in their child’s conditions. Doing so may help to reduce families’ anxieties and enhance trial recruitment and retention. (6) Parents may link treatment delays to the additional procedures required for the trial and this could discourage them from participating, or remaining, in the trial. Where possible, health professionals should avoid delays in delivering treatments pre and post-randomisation. This may also help to reduce families’ anxieties and improve trial recruitment and retention. (7) In cases where families’ treatment preferences conflict, randomisation may offer a means to resolve this conflict. Sensitively convey treatment arm allocation to families. If a child is upset with treatment allocation, exploring their anxieties and concerns about treatment may help to allay their concerns. Indeed, exploring and balancing treatment preferences pre-allocation could help prevent such difficulties, especially if a child is subsequently allocated to their non-favoured treatment and this is not available outside of the trial. Such discussions may help to avoid families withdrawing from the trial because they do not want to continue with the allocated treatment. If the child remains upset about the prospect of continuing with the treatment they have been allocated to, the opportunity of withdrawal and treatment options outside of the trial should be discussed. (8) Develop a strategy to allow families to indicate when they have made a decision regarding participation, so minimising delays from the perspective of families. This will help to reduce families’ anxieties about the condition progressing, avoid compromising their trust in health professionals and enhance trial recruitment. Future work should explore how best to implement such a strategy in time urgent settings. (9) Consider staffing strategies to support health professionals in recruiting families outside of normal working hours. (10) Avoid making statements to families that convey retrospective judgements about the suitability of a participant for one or other treatment arm after randomisation. Be aware of this particularly when discussing surgical findings with a trial participant's family after surgery. Explaining that non-operative treatment may have been inappropriate may deter their trust, which is a cornerstone of recruitment and retention in trials.	

### Strengths and limitations

Our analysis triangulated data on communication in CONTRACT consultations, with data on how this communication was experienced by children/young people, parents and health professionals across all CONTRACT sites. The qualitative sample was diverse and included those who participated and those who declined CONTRACT, and from both treatment groups. We obtained qualitative data for most families who were approached about CONTRACT, but it is possible that the consultations and views of families who did not take part in the Communication Study differed from those reported here. Nevertheless, the consultations and interviews we captured showed a range of approaches to CONTRACT communication and views about CONTRACT.

We qualitatively identified changes in communication behaviour in response to communication training. Although we also observed a quantitative increase in recruitment rates across the recruitment phases, a nested randomised controlled trial of recruitment training would be needed to infer that training increased trial recruitment rates.

## Conclusion

This qualitative study embedded within a paediatric feasibility trial demonstrated that delivering bespoke communication training to health professionals can enhance trial communication. Our analysis has informed a comprehensive list of recommendations that should be considered in developing a future definitive trial comparing non-operative treatment with appendicectomy in children with uncomplicated acute appendicitis. The recommendations can also be used to enhance informed consent and recruitment to other future paediatric trials, particularly in urgent care, surgical settings.

## Data Availability

The datasets used and analysed during the current study are not publicly available due to confidentiality but are available from the corresponding author on reasonable request.

## Abbreviation

CONTRACT: CONservative TReatment of Appendicitis in Children a randomised controlled Trial
